# A Pilot Study of Partial Unweighted Treadmill Training in Mobility-Impaired Older Adults

**DOI:** 10.1155/2014/321048

**Published:** 2014-02-19

**Authors:** Matthew J. Peterson, Nanyamka Williams, Kevin Caves, Miriam C. Morey

**Affiliations:** ^1^Geriatric Research, Education and Clinical Center, Veteran's Affairs Medical Center, Durham, NC 27710, USA; ^2^Aging Center/Pepper OAIC, Duke University, Durham, NC 27710, USA; ^3^Division of Geriatrics, Department of Medicine, Duke University, Durham, NC 27710, USA; ^4^Division of Speech and Audiology, Department of Surgery, Duke University Medical Center, Durham, NC 27710, USA; ^5^Pratt School of Engineering, Department of Biomedical Engineering, Duke University, Durham, NC 27708, USA

## Abstract

*Background*. Partial unweighted treadmill training is a potentially effective modality for improving fitness and function in frail elders. We tested the feasibility of partial unweighted treadmill training in older, mobility-impaired veterans. *Methods*. Eight mobility-impaired elders participated in partial unweighted treadmill training three times/week for twelve weeks. Outcome measures included gait speed, performance-oriented mobility assessment (POMA), eight foot up and go, and the SF-36 physical functioning short form. *Results*. There was significant improvement in treadmill walking time (+8.5 minutes; *P* < 0.001), treadmill walking speed (+0.14 meters/second; *P* = 0.02), and percent of body weight support (−2.2%; *P* = 0.02). Changes in physical performance included usual gait speed (+0.12 meters/second; *P* = 0.001), rapid gait speed (+0.13 meters/second; *P* = 0.01), POMA (+2.4 summary score; *P* < 0.001), and eight foot up and go (−1.2 seconds; *P* = 0.05). *Conclusions*. Partial unweighted treadmill training is feasible in mobility-impaired elders. Improvements in treadmill training capacity resulted in clinically meaningful improvements in fitness levels and improved mobility.

## 1. Introduction

Body weight supported treadmill training (BWSTT) has emerged over the last twenty years as a rehabilitation method focused on improving gait and function [1–10]. Reports of improved over-ground walking speed as a result of BWSTT are of particular interest, as impaired walking is a strong predictor of numerous adverse health outcomes [11–13]. We view certain components of BWSTT (principally partial unweighting) as potentially very effective in improving the fitness and function of slow and/or unsteady walking elders. In a clinical setting, over many years, we have observed slow walking elders avoid treadmill training due to perceived or real functional deficits. Therefore, partial unweighted treadmill training has potential to fill a clinical need for mobility-impaired participants in an outpatient exercise and health promotion program for senior veterans (see program description below). We hypothesize that even a minimal amount of unweighting can increase comfort and safety, decrease cardiovascular and musculoskeletal system demands, increase treadmill time tolerated, and subsequently improve fitness and function.

## 2. Materials and Methods 

### 2.1. Participants

Participants (*N* = 8) were active patients in Gerofit, an outpatient exercise and health promotion program at the Durham Veteran's Affairs Medical Center that has been described in detail [14, 15]. Entry into the Gerofit program requires VA primary provider approval, independence in activities of daily living (e.g., able to function independently in the program), and the ability to provide own transportation to and from the program. Additionally, exclusion criteria for Gerofit participation include unstable angina, proliferative diabetic retinopathy, oxygen dependence, incontinence, open wounds, active substance abuse or homelessness, and behavioral issues that preclude one from participating in a group setting.

The Gerofit program meets 3 days a week, with participants exercising for 60 to 90 minutes per session. A typical exercise session consists of 10 minutes of warm-up exercises, 20 to 40 minutes of aerobic exercise, 15 to 20 minutes of strengthening exercises, and 20 minutes of floor exercises designed to focus on musculoskeletal strengthening, flexibility, balance, and coordination. Participants were required to have been enrolled and actively participating in Gerofit for a minimum of six months to minimize physiologic training effects due to regular exercise. Study inclusion criteria also included either having a usual gait speed of less than 1.0 meter/second [12], or inability to walk safely on a treadmill as determined by Gerofit staff. This study was approved by the Durham VA Institutional Review Board, and all participants provided written consent prior to participation in the study.

### 2.2. Intervention

Partial unweighted treadmill training was conducted three days per week for twelve weeks. The initial weight support, treadmill speed, and walking time were individualized based on ability, and the training sessions were incorporated into the participants' Gerofit routine. The baseline functional assessment, in particular usual gait speed, was used as an indicator of initial treadmill walking speed for participants. For example, if over-ground gait speed was 0.60 meters/sec (~1.3 miles/hour), then the target initial treadmill walking speed was at or near this walking speed. The first training session was then devoted to acclimation to the treadmill and harness system and confirming a comfortable walking speed and percent body weight support for each participant. An exercise physiologist supervised all training sessions, and progressions throughout the intervention followed the guidelines of the American College of Sports Medicine [16]. The Borg rating of perceived exertion [17] was used as a measure of exercise intensity and monitored throughout the intervention. The Borg scale ranges from 6 (no effort) to 20 (maximal effort), with a range of 12–14 generally indicating moderate intensity activity. Participants were asked after approximately one to two minutes of walking, “How hard are you working right now?” Treadmill speed was increased or decreased accordingly, based on exertion levels above or below the target window of 12–14. Perceived exertion was then reevaluated every two to three minutes throughout their treadmill walking session with the goal of remaining at the 12–14 exertion level. The focus of progression occurred in the following order: (1) walking time increased by approximately 10%, (2) walking speed increased by approximately 10%, and (3) weight support decreased by approximately 10%. Increasing treadmill walking time was the first priority, as the intervention replaced the aerobic portion of the participants' Gerofit routine. If a participant was able to achieve twenty consecutive minutes of treadmill walking we then focused on increasing walking speed with a goal of 1.0 meters/second (~2.2 miles/hour). Changes in unweighting were given the lowest priority, as the amount required for safe and comfortable walking was minimal (~5-6%) from the onset.

### 2.3. Measures

#### 2.3.1. Health and Functional Measures

Usual and rapid gait speed was collected by measuring time taken to walk eight feet at a normal pace and as fast as possible. This test was administered using a digital stop watch and strictly followed the instructions specific to measuring gait speed as part of the Short Physical Performance Battery [18], which is a well validated test [19]. The eight foot up and go was administered and consists of rising from a chair, walk around a cone eight feet away, and return to a seated position in the chair as fast as safely possible [20, 21]. The performance-oriented mobility assessment (POMA) consists of stability tasks that are related to daily activities as well as a qualitative examination of locomotion pattern. The summary score has a range from 0 to 28 [22]. The SF-36 physical functioning questionnaire was used as a measure of self-reported health limitations. Scores range from 0 to 100 with a higher score indicating better function [23]. A modified version of the Older Americans Resources and Services (OARS) Comorbidity Scale ascertained self-reported prevalent health conditions and symptoms [24]. The original OARS questionnaire is a yes or no checklist of thirty-two comorbid conditions. We modified the questionnaire by adding eleven highly prevalent symptoms in older veterans, including pain, feeling tired, trouble sleeping, depression or memory problems, muscle weakness, dizziness, shakiness, balance problems, fear of falling, and numbness or tingling.

#### 2.3.2. Equipment

**Figure 1:**
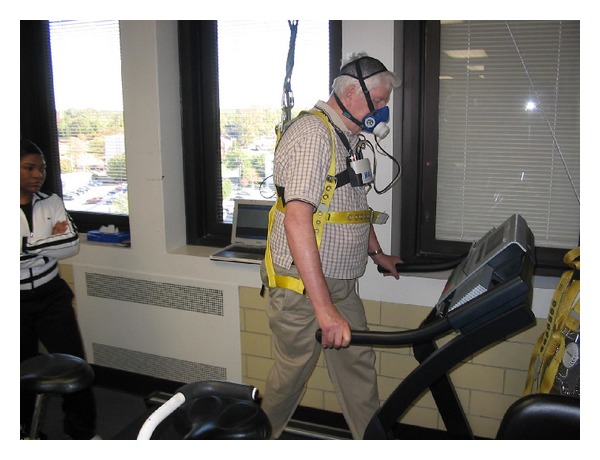
Unweighted treadmill training system (cardiorespiratory data is also being collected).

The treadmill pulley system was conceptualized, engineered, and assembled in-house by the study team. It essentially consisted of a climbing harness, which was belted around the participants' thighs and shoulders with adjustable straps (see Figure 1). The harness was then attached to an overhead pulley system. A livestock grade, zeroed scale was suspended from the pulley cable to allow measurement of weight that the pulley system was supporting. The scale was not able to measure dynamic changes in weight due to walking; however, this is a common limitation in commercially available weight-assisted systems. Weight support was adjusted via a manual winch. A secondary safety cable was secured in the unlikely event of a failure of the pulley system.

#### 2.3.3. Data Analysis

Baseline characteristics were examined using univariate procedures. The primary method of data analysis for repeated outcome measures was mixed models or hierarchical linear models, and baseline values for outcomes as well as number of diagnoses/symptoms were entered as covariates in all models. In these models, individual change is represented through a two-level hierarchical model. Significance was set at the *P* < 0.05 level, and all analyses were conducted using SAS v 8.3 statistical software (SAS, Cary, NC).

## 3. Results

The mean age of the participants was eighty-two years (Table 1). Usual gait speed was less than 1.0 meter/second for all but participant number 3, whose gait speed was 1.1 meters/second. This participant was eligible for the study because of his inability to safely treadmill walk due to early onset Parkinson's disease. Participant 6 dropped out of the study at seven weeks due to an injurious fall at home. This group had a high burden of disease and symptoms, with a mean of approximately eleven, indicating that this was a frail group as measured by the deficit accumulation index. The most prevalent diseases and symptoms at baseline are shown in Table 2. Hypertension, diabetes, and self-reported balance problems were reported in 56% of participants, while 50% reported hearing problems, heart trouble, muscle weakness, and a fear of falling. Arthritis or rheumatism was prevalent in 38% of participants.

### 3.1. Impact on Treadmill Walking Capacity

There was a significant improvement in treadmill walking speed, weight support required, and walking duration (Table 3). The mean treadmill training walking speed improved from 0.80 meters/second at week one to 0.94 meters/second at twelve weeks (*P* = 0.02). By study completion no one of the participants required weight support during treadmill training. At week one the mean treadmill training time was approximately eleven minutes, and by study completion all participants had achieved the study goal of treadmill training for twenty consecutive minutes (*P* < 0.001).

### 3.2. Impact on Functional Measures

Functional measures are reported in Table 4. The mean usual gait speed improved by 0.15 meters/second (*P* = 0.001) and rapid gait speed improved by 0.16 meters/second (*P* = 0.01) over twelve weeks. The mean POMA summary score improved by almost four points (21.1 to 24.9; *P* < 0.001), and the time to complete the eight foot up and go improved by 1.18 seconds (*P* = 0.05). There was not a significant improvement in self-reported physical functioning (50.6 to 52.9; *P* = 0.59).

## 4. Discussion

This pilot study of unweighted treadmill training in mobility-impaired elders yielded several important findings. Overall there was a 74% improvement in walking time during the intervention (11.5 to 20 minutes; *P* < 0.001), suggesting improved aerobic fitness levels of the participants. Another training goal was to progress to treadmill training at a minimum of 1.0 meters/second, and as a group this goal was nearly achieved. This walking speed has been shown to be a clinically important threshold for increased risk of functional loss and declining health [12, 25].

The improvement in usual gait speed (+0.15 meters/second; *P* = 0.001) is clinically very meaningful. Hardy and colleagues [26] reported that in Medicare and Veteran's Affairs older patients, an improvement in gait speed of ≥0.1 meters/second was associated with a 58% reduction in eight-year mortality rates, compared to those whose gait never improved. If the improvements in usual gait speed observed in the present study were maintained, it is plausible that similar survival benefits would be conferred. The mean rapid gait speed at baseline (1.17 meters/second) was slower than published norms [27] and was associated with increased risk for early cognitive decline [28]; however, the improvements observed over twelve weeks (+0.16 meters/second; *P* = 0.01) suggest an abatement of this risk.

At baseline, the mean POMA score of 21 suggested a moderate risk of falling [22]. However, at the study's completion the significant improvement in the mean POMA score to approximately 25 (*P* < 0.001) indicated that the group's risk of falling was low. Similarly, with the eight foot up and go test, the group's time to complete the test at baseline (9.3 seconds) was associated with an increased risk of falling (above nine seconds is the risk cut-off), and at twelve weeks (mean = 8.1 seconds; *P* = 0.05) that risk was no longer present [20, 21]. These changes are of considerable importance to this group, as two of their most prevalent self-reported conditions at baseline were balance problems (56%) and a fear of falling (50%).

Limitations to this study include having no control group and drawing from a sample of convenience, so generalizing these findings broadly to the community dwelling older adult population should be done with caution. Due to the exploratory nature of this pilot study we cannot speculate into mechanisms (i.e., physiological, biological, and psychological) that lead to observed improvements. Follow-up studies are planned to explore these and other potential mechanisms for change. Whether these changes have an impact on, or are mitigated by, health conditions or their symptoms is of particular interest to us. Another limitation is that the intervention and outcome measures were administered by same person. Lastly, the length of the intervention was minimal at twelve weeks.

## 5. Conclusions

This pilot study demonstrated the feasibility of unweighted treadmill training in mobility-impaired elders. The significant gains in treadmill training capacity resulted in clinically meaningful improvements in fitness over twelve weeks. More importantly, the magnitude of the group's improvements in over-ground gait, balance, and mobility was associated with lowered risk for a multitude of adverse health events.

## Figures and Tables

**Table 1:** Baseline characteristics of participants.

Participant	Age	Height (Cm)	Weight (Kg)	Usual gait speed (meters/second)	Diseases/symptoms
1	77	168.9	91.8	0.50	10
2	86	182.9	84.1	0.84	12
3	77	191.8	100.5	1.11	10
4	86	184.2	78.6	0.68	12
5*	79	162.6	89.5	0.81	13
6	88	166.4	66.6	0.87	14
7	81	180.3	83.1	0.84	6
8	82	176.5	80.5	0.94	8
Mean ± SD	82 ± 4	176.7 ± 10.0	84.4 ± 10.1	0.82 ± 0.18	10.6 ± 2.7

Cm: centimeters; Kg: kilograms; *subject 5 was the only female in the study; SD: standard deviation.

**Table 2:** Prevalent diseases/symptoms in study participants (*N* = 8) at baseline.

Disease/symptom	% with disease/symptom
Diabetes	56%
Hypertension	56%
Balance problems	56%
Hearing problems	50%
Heart trouble	50%
Muscle weakness	50%
Fear of falling	50%
Arthritis or rheumatism	38%

**Table 3:** Change in speed, percent weight support, and walking time over twelve weeks (*N* = 8).

	Week one	Six weeks	Twelve weeks	*P* value*
	Mean ± SD	Mean ± SD	Mean ± SD	
Walking speed (m/s)	0.80 ± 0.27	0.89 ± 0.27	0.94 ± 0.13	0.01
% of body weight supported	5.6 ± 2.8	3.4 ± 3.9	0	0.02
Walking time (min)	11.5 ± 4.3	18.0 ± 2.4	20.0 ± 0	<0.001

SD: standard deviation; m/s: meters per second; min: minutes; *for change over twelve weeks, controlling for baseline value of outcome variable and count of baseline diseases/symptoms.

**Table 4:** Change in functional measures over twelve weeks (*N* = 8).

	Baseline	Six weeks	Twelve weeks	*P* value*
	Mean ± SD	Mean ± SD	Mean ± SD	
Usual gait speed (m/sec)	0.82 ± 0.18	0.94 ± 0.14	0.97 ± 0.16	0.001
Rapid gait speed (m/sec)	1.17 ± 0.17	1.30 ± 0.21	1.33 ± 0.27	0.01
POMA summary (0–28)	21.1 ± 2.2	23.5 ± 1.3	24.9 ± 0.9	<0.001
Eight foot up and go (sec)	9.25 ± 1.96	8.78 ± 1.55	8.07 ± 1.39	0.05
SF-36 Phys. func. (0–100)	50.6 ± 18.0	55.0 ± 15.8	52.9 ± 18.2	0.59

SD: standard deviation; *for change over twelve weeks, controlling for baseline value of outcome variable and count of baseline diseases/symptoms.

## References

[1] Bogey R, Hornby TG (2007). Gait training strategies utilized in poststroke rehabilitation: are we really making a difference?. *Topics in Stroke Rehabilitation*.

[2] Sullivan KJ, Knowlton BJ, Dobkin BH (2002). Step training with body weight support: effect of treadmill speed and practice paradigms on poststroke locomotor recovery. *Archives of Physical Medicine and Rehabilitation*.

[3] Finch L, Barbeau H (1986). Hemiplegic gait: new treatment strategies. *Physiotherapy Canada*.

[4] Fisher BE, Wu AD, Salem GJ (2008). The effect of exercise training in improving motor performance and corticomotor excitability in people with early Parkinson’s disease. *Archives of Physical Medicine and Rehabilitation*.

[5] Hicks AL, Martin Ginis KA (2008). Treadmill training after spinal cord injury: it’s not just about the walking. *Journal of Rehabilitation Research & Development*.

[6] Lo AC, Triche EW (2008). Improving gait in multiple sclerosis using robot-assisted, body weight supported treadmill training. *Neurorehabilitation & Neural Repair*.

[7] Mossberg KA, Orlander EE, Norcross JL (2008). Cardiorespiratory capacity after weight-supported treadmill training in patients with traumatic brain injury. *Physical Therapy*.

[8] Pohl M, Mehrholz J, Ritschel C, Rückriem S (2002). Speed-dependent treadmill training in ambulatory hemiparetic stroke patients: a randomized controlled trial. *Stroke*.

[9] Vidoni ED, Tull A, Kluding P (2008). Use of three gait-training strategies in an individual with multiple, chronic strokes. *Journal of Neurologic Physical Therapy*.

[10] Hoyer E, Jahnsen R, Stanghelle JK, Strand LI (2012). Body weight supported treadmill training versus traditional training in patients dependent on walking assistance after stroke: a randomized controlled trial. *Disability and Rehabilitation*.

[11] Guralnik JM (1994). Understanding the relationship between disease and disability. *Journal of the American Geriatrics Society*.

[12] Studenski S, Perera S, Wallace D (2003). Physical performance measures in the clinical setting. *Journal of the American Geriatrics Society*.

[13] Purser JL, Weinberger M, Cohen HJ (2005). Walking speed predicts health status and hospital costs for frail elderly male veterans. *Journal of Rehabilitation Research & Development*.

[14] Morey MC (2007). Celebrating 20 years of excellence in exercise for the older veteran. *Federal Practitioner*.

[15] Morey MC, Crowley GM, Robbins MS, Cowper PA, Sullivan RJ (1994). The Gerofit program: a VA innovation. *Southern Medical Journal*.

[16] American College of Sports Medicine (2009). *ACSM's Guidelines for Exercise Testing and Prescription*.

[17] Borg G (1998). *Borg's Perceived Exertion and Pain Scales*.

[18] Guralnik JM, Seeman TE, Tinetti ME, Nevitt MC, Berkman LF (1994). Validation and use of performance measures of functioning in a non-disabled older population: MacArthur studies of successful aging. *Aging*.

[19] Guralnik JM, Ferrucci L, Pieper CF (2000). Lower extremity function and subsequent disability: consistency across studies, predictive models, and value of gait speed alone compared with the short physical performance battery. *The Journals of Gerontology A *.

[20] Rikli RE, Jones CJ (1999). Development and validation of a functional fitness test for community-residing older adults. *Journal of Aging and Physical Activity*.

[21] Rikli RE, Jones CJ (1999). Functional fitness normative scores for community-residing older adults, ages 60–94. *Journal of Aging and Physical Activity*.

[22] Tinetti ME (1986). Performance-orientated assessment of mobility problems in elderly patients. *Journal of the American Geriatrics Society*.

[23] Ware JE, Sherbourne CD (1992). The MOS 36-item short-form health survey (SF-36). I. conceptual framework and item selection. *Medical Care*.

[24] Fillenbaum GG, Maddox G (1987). OARS multidimensional functional assessment questionnaire. *The Encyclopedia of Aging*.

[25] Cesari M, Kritchevsky SB, Penninx BWHJ (2005). Prognostic value of usual gait speed in well-functioning older people—results from the health, aging and body composition study. *Journal of the American Geriatrics Society*.

[26] Hardy SE, Perera S, Roumani YF, Chandler JM, Studenski SA (2007). Improvement in usual gait speed predicts better survival in older adults. *Journal of the American Geriatrics Society*.

[27] Bohannon RW (1997). Comfortable and maximum walking speed of adults aged 20–79 years: reference values and determinants. *Age and Ageing*.

[28] Fitzpatrick AL, Buchanan CK, Nahin RL (2007). Associations of gait speed and other measures of physical function with cognition in a healthy cohort of elderly persons. *The Journals of Gerontology A*.

